# Intrafascial Colpotomy, Edge-to-Edge Closure, and Peritoneal Graft Technique for Minimizing Mesh Erosion in Concurrent Robotic Hysterectomy and Sacrocolpopexy

**DOI:** 10.1007/s00192-024-06012-x

**Published:** 2024-12-05

**Authors:** Yael Yagur, Assem Kalantan, Mujahid Bukhari, Orla Donohoe, Mohammed Almoqren, Jessica Robertson, Sarah Choi, David Rosen, Zhuoran Chen, Kate Moore, Danny Chou

**Affiliations:** 1 Sydney Women’s Endosurgery Centre (SWEC), St George Private Hospital, Kogarah, Sydney, NSW Australia; 2 University of New South Wales, Sydney, Australia; 3 Pelvic Floor Unit, St George Hospital, Kogarah, Sydney, Australia; 4 Department of Obstetrics and Gynecology, Meir Medical Center, Kfar Saba, Israel; 5 School of Medicine, Faculty of Medical and Health Sciences, Tel Aviv University, Tel Aviv, Israel

**Keywords:** Mesh erosion, Pelvic organ prolapse (POP), Peritoneal graft, Robotic hysterectomy, Sacrocolpopexy (SCP)

## Abstract

**Introduction and Hypothesis:**

Sacrocolpopexy (SCP) is a recognized treatment for apical pelvic organ prolapse (POP). However, mesh erosion remains a concern, particularly when performed with concomitant hysterectomy. This video presents data on one case of a modified technique aimed at potentially minimizing mesh erosion in robotic SCP.

**Methods:**

This technique focuses on reinforcing the vaginal cuff and using a pedicled peritoneal graft to create a tissue barrier between the mesh and the vaginal vault. Procedural steps include intrafascial colpotomy, edge-to-edge cuff closure using barbed sutures, and joining anterior and posterior meshes away from the vaginal cuff.

**Results:**

The surgical technique was successfully implemented in this single patient presented in the video and was performed in ten more patients with no intraoperative or postoperative complications. During the follow-up period, there were no signs of mesh erosion or exposure.

**Conclusions:**

This approach emphasizing vaginal cuff strengthening and mesh separation using a pedicled peritoneal graft can be an option for reducing mesh erosion risk. This report does not provide definitive evidence that this approach reduces mesh erosion risk and further research and long-term follow-up are required to validate these findings and integrate this technique into standard management practices.

**Supplementary Information:**

The online version contains supplementary material available at 10.1007/s00192-024-06012-x.

## Introduction

Sacrocolpopexy (SCP) is a surgical procedure for the management of apical pelvic organ prolapse (POP) [1] and can be performed concurrently with a total hysterectomy in the same operation to decrease the risk of recurrence and eliminate the need for future management of uterine and cervical pathologies [2–4]. Rates of SCP for apical vaginal prolapse vary depending upon surgeon preference and technical ability with a reported prevalence of 17.5% of POP surgeries in the United States [5]. The technique involves anchoring a synthetic mesh to the anterior and posterior walls of the vagina and securing it to the anterior longitudinal ligament of the sacrum to provide pelvic support [6]. Minimally invasive SCP is widely recognized as one of the most effective treatments for POP, offering a lower recurrence rate compared to alternative surgical approaches [7].

However, the use of mesh in this procedure alone or with concomitant total hysterectomy has been associated with erosion and exposure [8]. Reported rates of mesh erosion range from 0% to 7.7% [9–11]. Refining surgical techniques to minimize these risks should be considered to reduce pain, infection rates, injury to adjacent organs, and the need for additional corrective surgery. In this innovative video, we present surgical technique aims to potentially protect the vaginal vault at SCP with concomitant hysterectomy, thereby reducing the risk of vault mesh erosion.

## Methods

The fundamental elements of this method are the strengthening of the vaginal cuff and interposition of a barrier between the mesh and vaginal vault epithelium using a pedicled peritoneal graft harvested from the uterovesical fold. A further measure involved joining the anterior and posterior meshes away from the vaginal cuff itself.

**Fig. 1:**
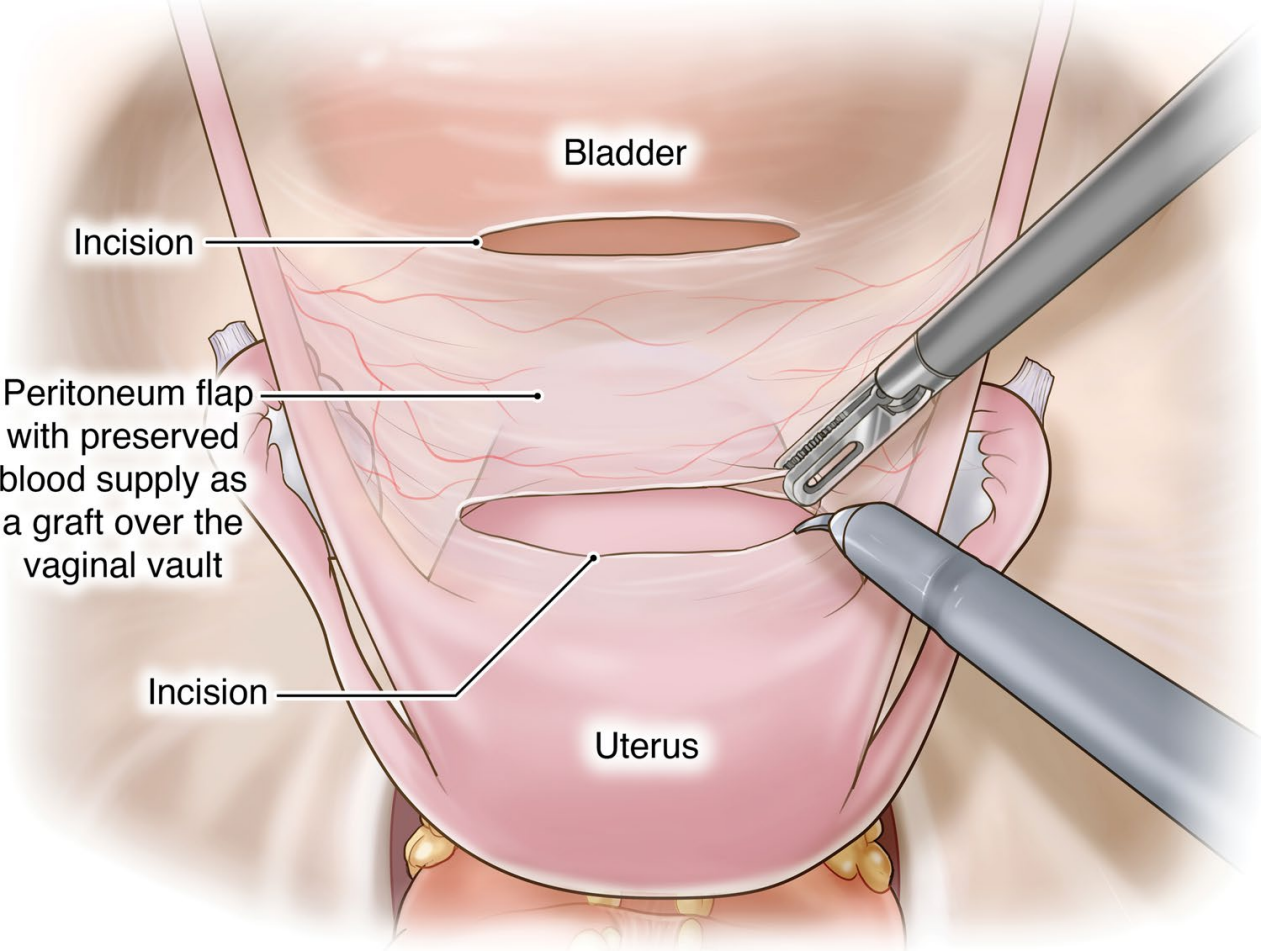
Creation of the pedicled peritoneal graft
  **Fig. 2:**
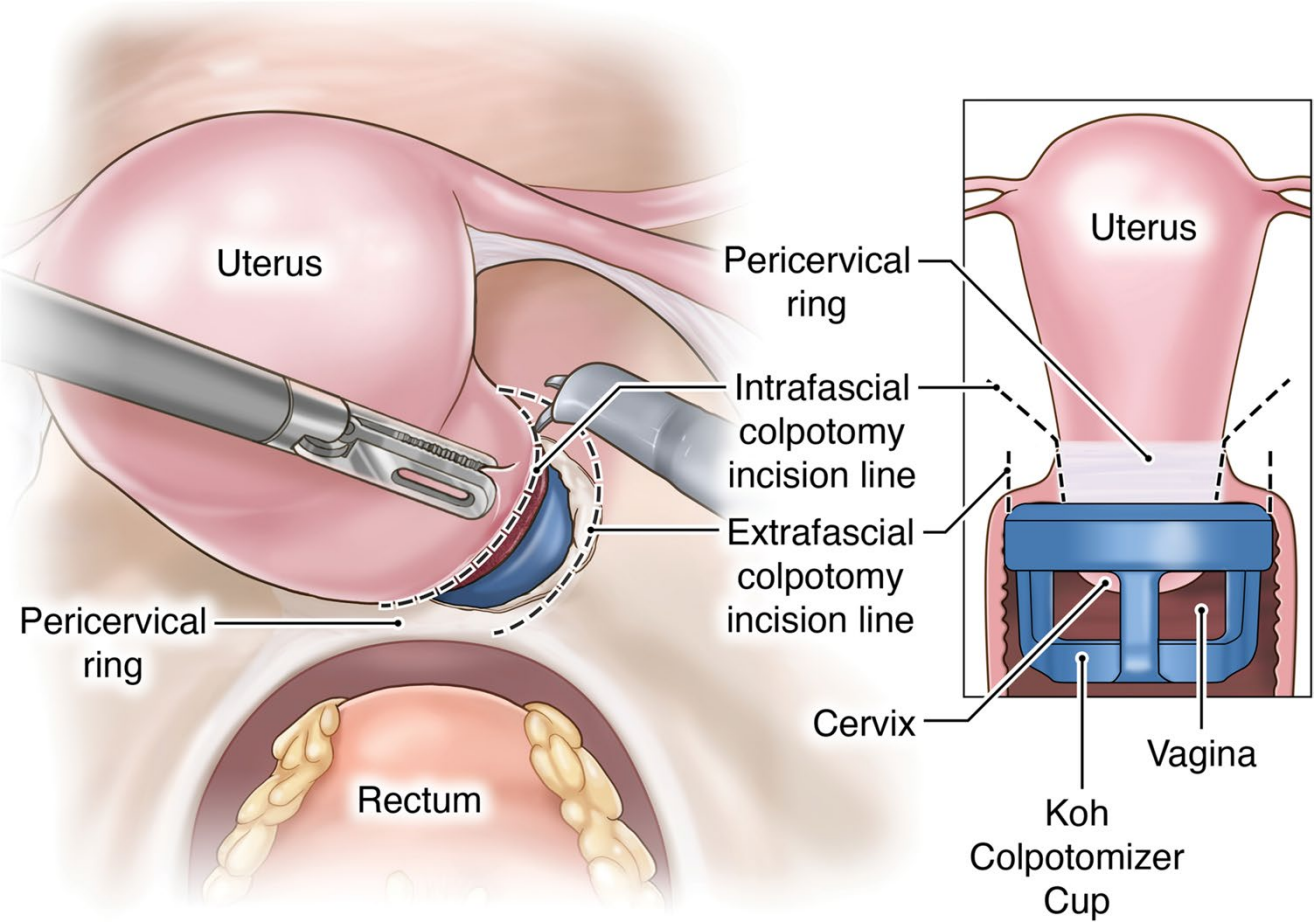
Infrafascial colpotomy
  **Fig. 3:**
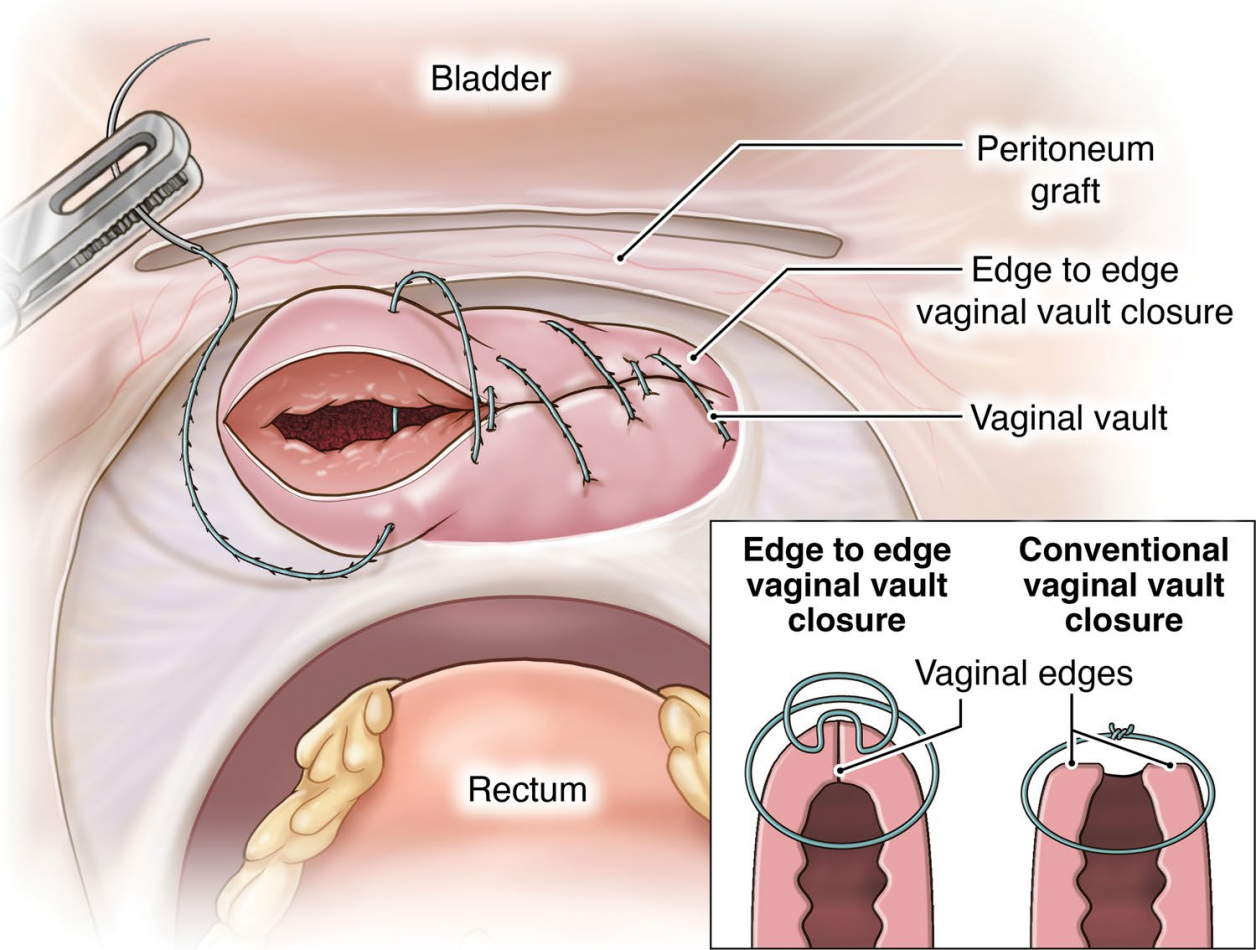
Edge to edge closure of the cuff
  **Fig. 4:**
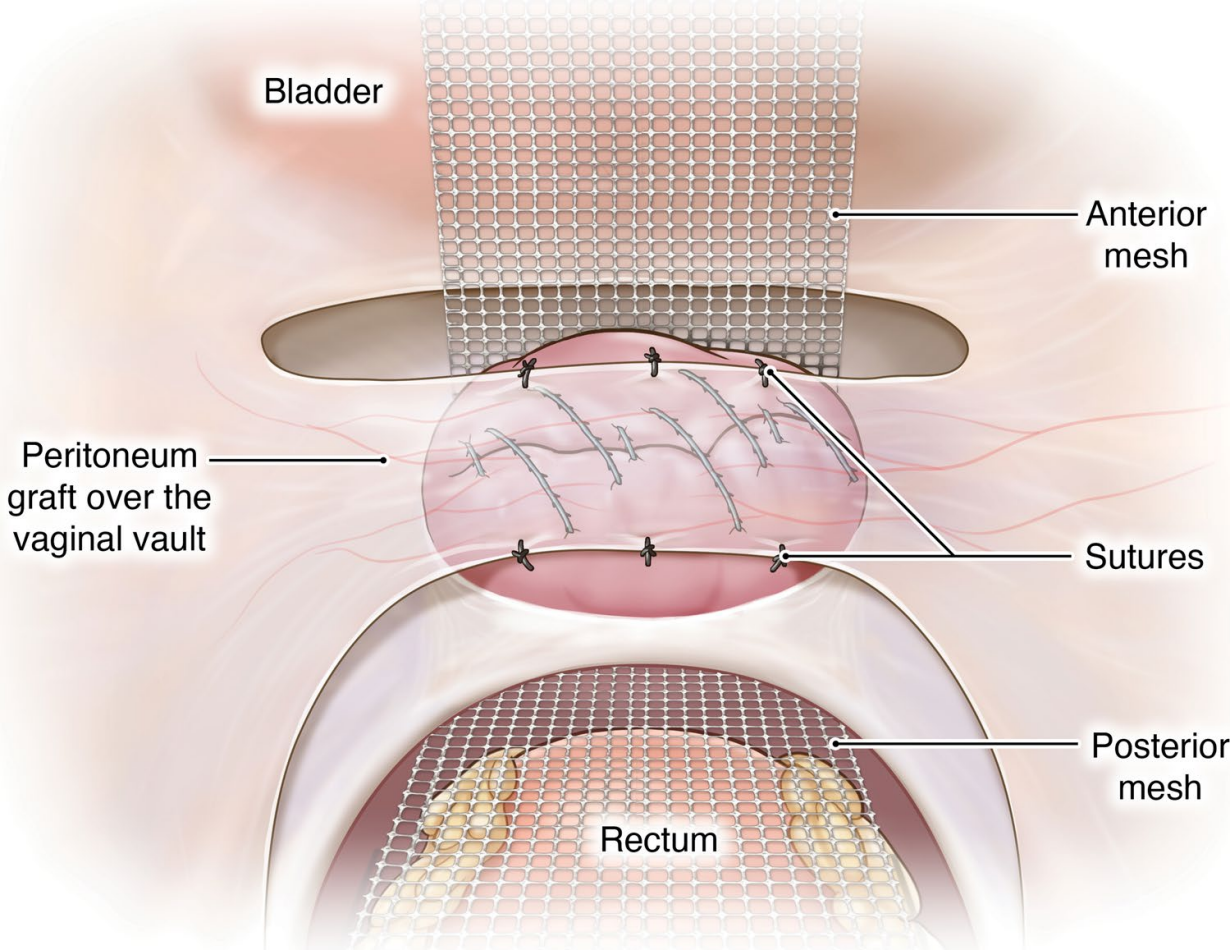
Peritoneal graft placement on the vaginal vault
  **Fig. 5:**
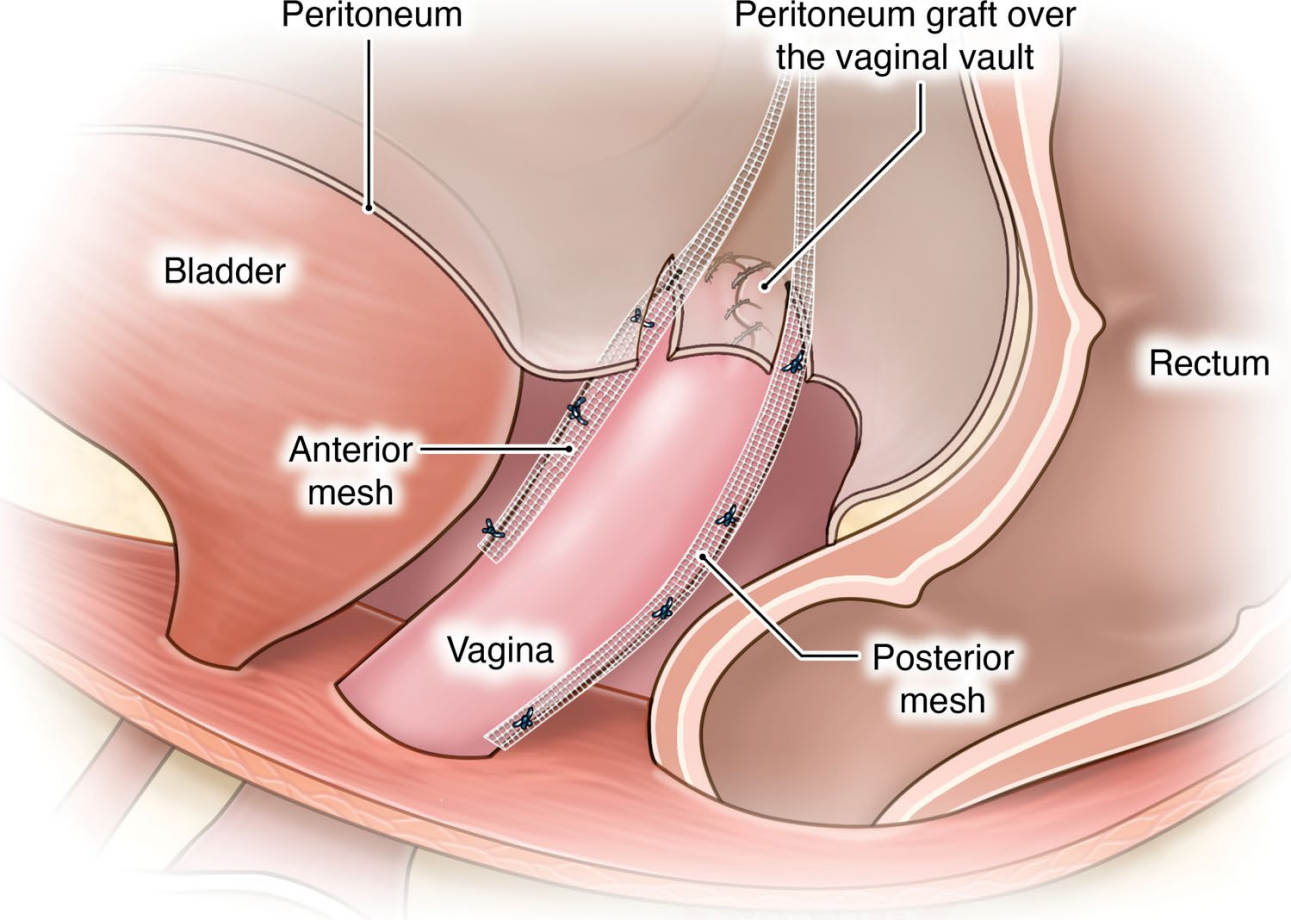
Joining of the mesh away from the vaginal vault

The proposed technique involves unique procedural steps:

1. **Creation of the pedicled peritoneal graft**: This process includes making two horizontal incisions on the uterovesical fold peritoneum. These incisions are spaced 3 cm apart to ensure that an adequate length and width of peritoneal tissue can be harvested for the graft (Fig. 1).
2. **Intrafascial colpotomy**: Following the normal steps of robotic hysterectomy, an intrafascial colpotomy is performed starting at halfway between the uterine isthmus and cervicovaginal junction preserving the pubocervical fascia and uterosacral ligaments. This critical step results in a much thicker vaginal cuff through the preservation of the pubocervical fascia and uterosacral ligaments. This thicker vaginal cuff makes it possible to suture in edge-to-edge fashion with resultant thick vaginal vault that would be resilient to mesh erosion (Fig. 2).
3. **Edge-to-edge closure of the cuff**: Barbed 0 sutures are placed in an alternating manner, capturing the full thickness of the vaginal vault, followed by a superficial suture that brings the fascial edges together. This technique creates a thick and strong vaginal apex, reducing the risk of cuff dehiscence. The barbed sutures minimize tension along the suture line, promoting better healing. By incorporating the pubocervical fascia, rectovaginal septum, and uterosacral ligaments into the closure, the risk of future prolapse is further reduced. Additionally, this closure provides an added protective layer between the mesh and the vaginal epithelium (Fig. 3).
4. **Peritoneal graft placement on the vaginal vault**: The pedicled peritoneal graft is sutured to the vaginal vault with interrupted sutures to interpose between the vaginal vault and the mesh graft (Fig. 4).
5. **Joining of the mesh away from the vault**: The individually tailored anterior and posterior meshes are joined together after suturing to the anterior and posterior vaginal wall at least 3 cm away from the vaginal cuff to avoid direct contact over it (Fig. 5).

## Results

We are presenting this as a single-case video, and we have since performed the technique in ten additional patients. The proposed surgical technique was successfully performed, and discharge occurred as planned. No intraoperative complications were noted. No postoperative complications were observed, and the postoperative course was uneventful. During the follow-up period, no signs of mesh erosion or exposure have been observed to date.

## Conclusions

SCP has long been recognized as an effective treatment for POP [1]. Mesh-related complications can adversely affect patient outcomes and may require additional surgeries, thereby increasing morbidity [8].

This proposed surgical technique is offered as a potential approach to minimize the risk of mesh erosion during concomitant robotic hysterectomy with SCP. The key aspects of our approach include reinforcing the vaginal cuff and interposing a peritoneal graft between the mesh and the vaginal vault epithelium. Further research and longer-term follow-up are needed to assess these preliminary outcomes and evaluate the potential role of this technique in the surgical management of POP.

## Supplementary Information

Below is the link to the electronic supplementary material.

Supplementary file1 (MP4 90670 KB)
